# Registration of Feature-Poor 3D Measurements from Fringe Projection

**DOI:** 10.3390/s16030283

**Published:** 2016-02-24

**Authors:** Sebastian von Enzberg, Ayoub Al-Hamadi, Ahmed Ghoneim

**Affiliations:** 1 Institute for Information Technology and Communications (IIKT), Otto-von-Guericke University, PO Box 4120, 39016 Magdeburg, Germany; ayoub.al-hamadi@ovgu.de; 2 Department of Software Engineering, College of Computer Science and Information Sciences, King Saud University, Riyadh 11451, Saudi Arabia; ghoneim@ccis.ksu.edu.sa

**Keywords:** optical 3D sensor, stereo vision, 3D registration, surface measurement, fringe projection

## Abstract

We propose a novel method for registration of partly overlapping three-dimensional surface measurements for stereo-based optical sensors using fringe projection. Based on two-dimensional texture matching, it allows global registration of surfaces with poor and ambiguous three-dimensional features, which are common to surface inspection applications. No prior information about relative sensor position is necessary, which makes our approach suitable for semi-automatic and manual measurement. The algorithm is robust and works with challenging measurements, including uneven illumination, surfaces with specular reflection as well as sparsely textured surfaces. We show that precisions of 1 mm and below can be achieved along the surfaces, which is necessary for further local 3D registration.

## 1. Introduction

The increasing accuracy and speed of sensors for three-dimensional (3D) surface measurement allowed a number of novel inspection tasks to emerge in the last decade. Along with advanced signal processing, this led to the automation of surface inspection for quality and process control in industrial manufacturing, which previously relied on manual work by trained auditors. Also higher precisions allows quality control systems to meet the increasing demands and expectations of customers [1,2,3]. One such example is surface quality control of stamped sheet metal parts in the automobile industry, which requires a depth resolution of 10 µm to detect dents, which are barely visible on unpainted parts, but are revealed later in reflection patterns on the painted surface [4]. The goal is thus to detect these kind of surface defects in an early production stage, ideally after stamping in a sheet metal pressing plant. Active photogrammetric measurement systems using fringe projection are commonly used for this task, since they allow fast and precise non-contact acquisition of dense point clouds on homogeneous surfaces [5]. The measurement area of these systems is defined by the cameras’ field of view. Hence, the acquisition of data for large scale objects at consistent precision is not possible within a single measurement.

Numerous methods for registration of multiple overlapping range measurements are available (see e.g., [6]), however they either require an approximate global orientation or distinct 3D features for unique identification of points on different point clouds [7]. In surface quality inspection, the goal is to find slightest dents and bumps. The shape of objects under test is thus mostly flat and smooth, containing little curvature and few or even ambiguous 3D features. Their surface texture is also mostly homogenous, containing few points or areas of distinction. We refer to the class of surfaces with few distinct 2D and 3D features as *feature-poor*.

**Figure 1:**
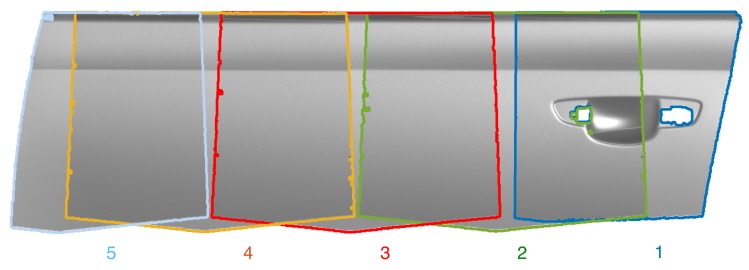
Visualisation of 5 partially overlapping measurements for a surface measurement of an unpainted sheet metal car door panel.

**Figure 2:**
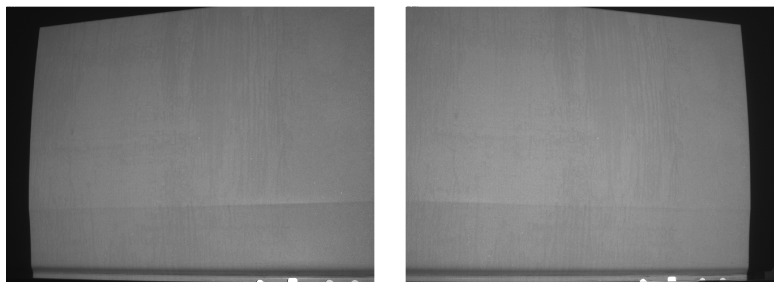
Left and right camera views for the measurement patch of a sheet metal car door panel.

One such example is given in Figure 1. The visualized car door segment results from measurement of 5 overlapping patches of a raw sheet metal part before assembly. The registration of the patches, especially number 3, 4 and 5, based solely on their 3D data is a challenging task. Also the stamped aluminium appears to have mostly homogenous texture when observed by the cameras of the measurement system, as can be seen in Figure 2. In controlled automated environments (e.g., with the sensor attached to a robot) approximate position information may be available. However, in semi-automatic or manual setups where prior knowledge is not available, correct registration would fail with current methods.

In this paper we present an approach for registration of 3D measurements based on surface texture which is extracted from the sensor’s camera images, eliminating the need of further hardware or prior knowledge of sensor position. Our main contribution is the formulation of a combined 2D/3D registration framework. It utilizes the most relevant information of surface texture directly from 2D camera images as well as geometric information from the 3D measurement. It thus works on surfaces with little curvature and few features, where pure 3D-based registration fails, and also if no further information about the sensor position is available. It is also robust to ambiguous matches by using RANSAC.

In Section 2, we will present the current state of the art in 3D surface measurement and 3D registration. We will present our texture-based registration algorithm in Section 3 and experimental results in Section 4. Our paper is finally concluded in Section 5.

## 2. Measurement and Registration of 3D Data

### 2.1. 3D Measurement for Surface Quality Inspection

**Figure 3:**
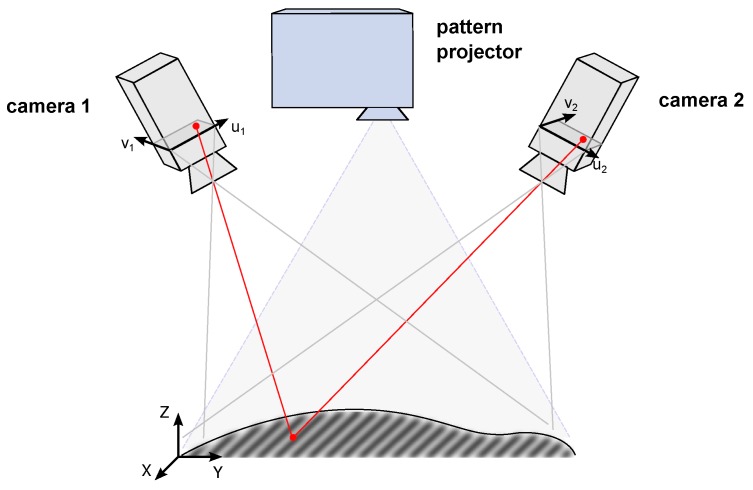
Triangulation of a 3D point (red) for a stereo camera sensor with pattern projection.

Among the wide range of possible setups (see [8] for a comprehensive review), a typical 3D measurement system with fringe projection consists of one or more digital cameras with known calibration [9] and a digital projector. When using a stereo or multi-camera setup, a 3D point can be computed by triangulation of the corresponding image points with known camera calibration (see Figure 3). This poses two main problems to a photogrammetric measurement system (among others): Finding corresponding image points in multiple camera views and camera calibration.

#### 2.1.1. Finding Corresponding Image Points

So called *passive photogrammetric systems* use unique surface texture for correlation, e.g., using image matching or using interest operators for matching of feature points [10]. For close-range application, *active* systems are commonly used, where a structured pattern is projected onto the surface. This allows the acquisition of much denser point clouds, higher measurement precision, and faster computation. Salvi [11] gives a review of commonly used patterns.

One such approach is the phase shift method [12], where a sinusoidal grating is projected onto the surface and shifted in equal intervals. The resulting image series allows accurate interpolation of phase values up to 1/100th of a pixel. Typical resulting sensor precision is 10 µm in range on a millimeter-grid for a measurement area of approximately 0.5 m by 0.5 m.

The texture matching of our registration algorithm operates on pairs or sets of images from the sensor’s cameras taken with uniform lighting. This can be achieved by *blank projection*, using the projector as a uniform light source without any pattern, and is part of many measurement methods anyway. If enough ambient lighting is available, an image can be taken with the projector completely deactivated; however in this case the camera integration time might need to be adjusted to obtain sufficient exposure of the surface. Most fringe projection sensors can thus be extended with our registration algorithm. The prototype sensor used in our experiments is equipped with a general purpose digital projector used for projection of a phase shift pattern series.

#### 2.1.2. Camera Calibration

Camera Calibration refers to a camera projection model and its parameters, as well as the process of determining the parameters. As a calibrated camera allows the formulation of a corresponding ray of light in 3D space for each image point, it is essential for *triangulation* of 3D points. The intersection point, or rather minimal distance point of the rays of two cameras’ corresponding image points leads to a 3D coordinate measurement. This constitutes the main principle of photogrammetric 3D measurement.

Most camera calibrations are based on a pinhole camera model. The resulting central projection can be described by the colinearity equation [13] with external parameters (*i.e*., camera translation and rotation) and internal parameters (focal length and principal point). Popular methods (e.g., Tsai [14] or Zhang [15]) elevate this by a model for radial, tangential and other lens distortions. Alternative camera models exist, which avoid explicit formulation of physically based projection parameters [16].

The calibration process consists of observing a scene with known 3D points or parameters and identifying corresponding 2D image points. The resulting over-determined system of projection equations can then be solved for the unknown calibration parameters. Common calibration rigs consist of one or multiple planes with well-defined patterns (e.g., checkerboard) or fiducial markers [17].

As with the projected pattern, our method is not restricted to a specific calibration method, the proposed registration can be easily applied to any 3D sensor with calibrated cameras that match the abstract formulation given in Section 3. The formulation of the calibration model used in our work can be found in Appendix.

Besides accurate camera calibration, low lens distortion, and camera noise, the measurement precision is mainly dependent on camera resolution. For a given image resolution, the range resolution, lateral resolution, and measurement area are typically interchanged to find an optimum for the application at hand. Without compromising measurement resolution, an increase of measurement area is only possible by increasing image resolution. This is often not possible due to increasing cost of the sensor and decreasing image quality. The only viable option are multiple measurements from different views with consecutive registration and merging into one combined measurement.

### 2.2. Registration of 3D Data

Registration of 3D range data is used in a wide scope of applications [18]. *Rigid registration* is the determination of the 6 translation and rotation parameters for a *source* surface such that the resulting transformed point cloud best matches a *target* surface. With a minimum of three known corresponding, non-colinear 3D points in the *source* and *target* surface, the transformation parameters can be computed via closed-form solutions [19]. Finding corresponding 3D points can be solved by adding special fiducial markers to the scene (target based registration) or by defining local interest points resulting from surface features [20]. For feature-poor surfaces, interest point descriptors as e.g., the popular intrinsic shape signature [21] fail to uniquely characterize 3D points. Also mounting of markers onto surfaces is not desired or possible in most close-range applications.

A key algorithm which uses dense point cloud information is Iterative Closest Point (ICP), originally presented by Besl/McKay [22]. Here, a local registration is iteratively refined by minimizing the euclidean distance error between nearest neighbour points. Increased accuracy can be achieved by using a point-to-plane distance measure [23] or even a plane-to-plane distance [24] instead of the original distance of neighbouring points. Another improvement comes from point correspondence rejection. Rejection can be based on a fixed or variable distance threshold, on a surface normal orientation threshold or statistically based (e.g., worst-percentage-rejection). See [6] for an overview of various improvements to the ICP algorithm. In general, ICP converges slowly and is susceptible to local minima. In order to find a global minimum, a good approximation of transformation parameters has to be known. Also, a robust registration is possible only if the source point cloud considered for registration is a subset of the target point cloud [25]. Usually point clouds are partly overlapping, so this is not a valid assumption; however with a reasonable correspondence rejection method as well as a good initial approximation of the transformation, a global solution can be found. If no approximation is known, some kind of point feature description has to be used. Blunders can then be effectively rejected by applying the RANSAC algorithm [26,27]. As stated above, these methods fail for feature-poor point clouds.

Alternatively to ICP, the registration problem can be treated as a least squares matching of the overlapping component of 3D surfaces [28]. This leads to a significantly lower number of iterations, slightly better registration accuracy and a more flexible algorithm, which can be easily extended to include statistical point error models or non-rigid registration parameters. However, the problems resulting from a missing initial solution and feature-poor surfaces are generally similar.

Colour or texture data has been previously used to enrich 3D data (see e.g., colour ICP [29]) and is usually dealt with as an additional data dimension to the surface measurement. Existing methods are then applied to the high dimensional point cloud (see e.g., [30,31,32]). These approaches however do not provide sufficient accuracy with regards to the texture information given. Due to triangulation errors, the mapping between image pixels and 3D points is imprecise. Also, the resolution of the 3D point cloud dictates the corresponding 2D texture resolution, which is usually less than the available camera resolution. Mapping texture data from 2D pixels to 3D points will thus lead to loss of information. This is critical for delicate and sparse texture, as it is present in feature-poor data given in surface inspection applications.

## 3. Texture-Based Registration Algorithm

Input to our proposed algorithm is the raw sensor data acquired by the structured-light system as well as the 3D data calculated thereof, namely:

**(a)**  **Camera calibration**
  (1) $$\left(u_{c},v_{c}\right)=f_{c}\left(x_{i},y_{i},z_{i}\right)$$
  gives a mapping from a 3D point in the sensor coordinate system $\left(x_{i},y_{i},z_{i}\right)$ of measurement *i* to a corresponding camera image coordinate $\left(u_{c},v_{c}\right)$ of camera *c*. Camera calibration is constant for each measurement *i*. Any model that matches this abstraction, can be considered. An example calibration model is given in Appendix. In the algorithm description below, we consider registration of two measurements i∈{1,2}, each taken with a stereo camera system c∈{1,2}.
**(b)**  **Camera image (blank projection)**
  (2) $$G_{i,c}\left(u_{c},v_{c}\right)$$
  the image $G_{i,c}$ typically is a matrix of (8-bit) integer gray scale values. Sub-pixel values for general image coordinates $\left(u_{c},v_{c}\right)$ result from bilinear interpolation of the four neighbouring pixels’ values. Again, each measurement is indicated by index *i* and each camera is indicated by index *c*.
**(c)**  **3D point cloud**
  (3) $$P_{i}=\left\{p_{j,i}\in R^{3}\;|\;j=1,2,...N_{i}\right\}$$
  where each 3D point $p_{j,i}$ results from triangulation of corresponding image points. Each measurement *i* may contain a different number $N_{i}$ of 3D points.

The algorithm output consists of a rotation matrix $R_{i}$ and translation vector $t_{i}$ for each measurement, which transform the point clouds from their respective sensor coordinate system into a common world coordinate system (X,Y,Z), according to

(4) $$P_{i}^{\prime }=R_{i}\;\cdot \;P_{i}+t_{i}$$

Processing is divided into three stages: extraction of texture from the camera images; identifying 2D keypoints from texture images and finding matches among measurements; and a robust estimation of 3D parameters that minimizes texture differences. The main challenge for the image processing is uneven illumination caused by the projector and specular surface reflection, as well as very sparse texture on the surface.

### 3.1. Texture Image

**Figure 4:**
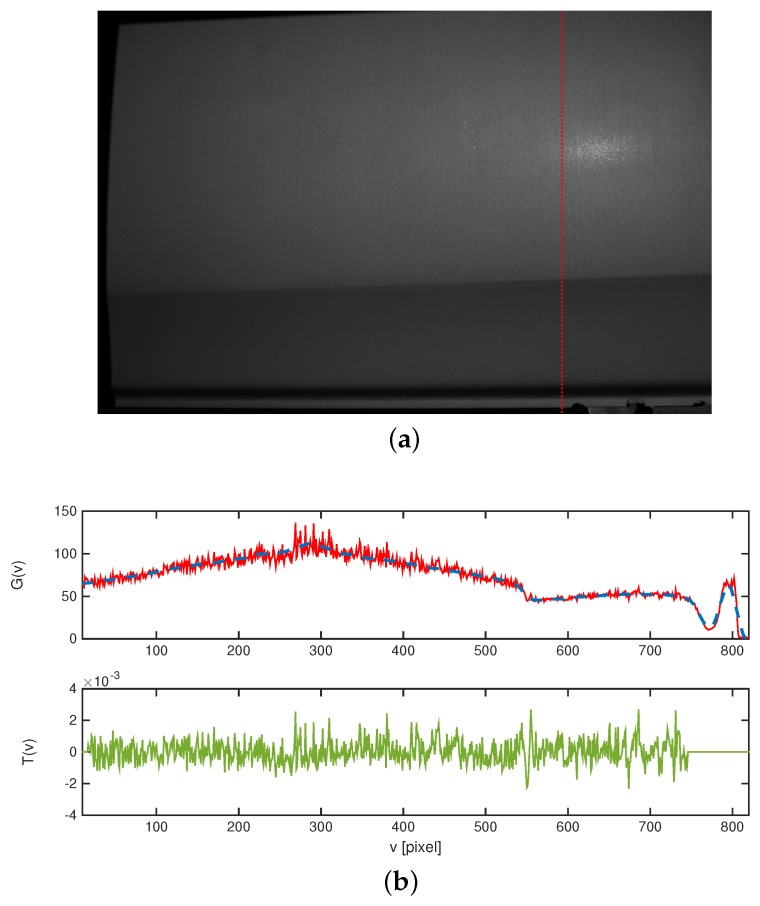
Image data of an unpainted car door panel (see measurement 3, Figure 1). (**a**) Measurement image (blank projection) of an unpainted car door panel; (**b**) Top: Gray scale sequence $G_{i,c}$ (red) and according filtered sequence $F_{i,c}$ (blue, dashed) along one image column. Bottom: resulting normalized, cropped texture $T_{i,c}$ (green) along the image column.

Texture extraction is composed of a filtering and a masking step. Figure 4a shows a typical bright image for a surface patch of an anodized aluminum sheet metal part used for a car body panel. Due to the specular reflective properties of the surface and the arrangement of camera and pattern projector, the surface is imaged with very uneven lighting. The effects of illumination as well as surface reflection can be approximated by low-pass filtering of the original image. This is visualized in the upper plot of Figure 4b, where a sequence of gray scale values $G_{i,c}\left(u_{c}^{0},v_{c}\right)$ along a single image column $u_{c}=u_{c}^{0}$ is shown alongside its low-pass filtered data $F_{i,c}\left(u_{c}^{0},v_{c}\right)$.

The objective of the filtering step is to generate an image ${\tilde{T}}_{i,c}\left(u_{c},v_{c}\right)$, which is widely independent of lighting and local surface curvature, with an approximatively uniform gray scale range along all parts of the resulting image. This can be realised by substracting and normalizing with the filtered image $F_{i,c}$, effectively attenuating bright image regions and amplifying dark regions.

(5) $${\tilde{T}}_{i,c}\left(u_{c},v_{c}\right)=\left|\frac{G_{i,c}\left(u_{c},v_{c}\right)-F_{i,c}\left(u_{c},v_{c}\right)}{F_{i,c}^{2}\left(u_{c},v_{c}\right)}\right|$$

In a subsequent masking step, the image is cropped to meaningful areas, by element-wise multiplication with an image mask $M_{i,c}$:

(6) $$T_{i,c}\left(u_{c},v_{c}\right)={\tilde{T}}_{i,c}\left(u_{c},v_{c}\right)\circ M_{i,c}\left(u_{c},v_{c}\right)$$

This is crucial for the correct identification and matching of key points in the next stage. The mask $M_{i,c}$ should exclude overly dark and overdriven areas as well as regions close to object borders. This can be achieved by applying a simple gray value threshold close to the limits of the gray scale range, followed by morphological erosion of the resulting binary mask with a square structuring element.

**Figure 5:**
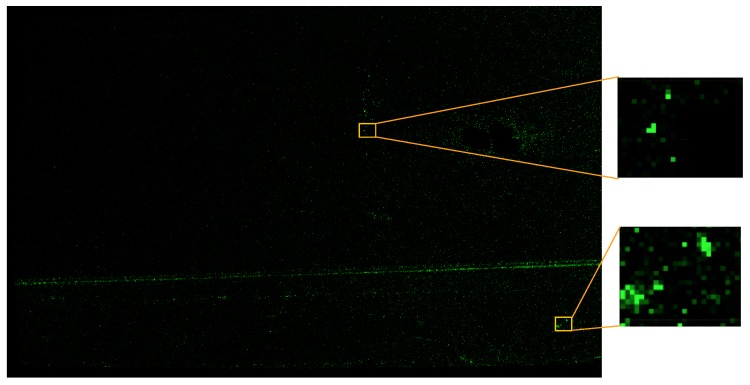
Resulting texture image *T* with zoomed texture details.

The resulting final texture data $T_{i,c}$ is displayed on the bottom of Figure 4a for one image column and in Figure 5 for a complete image.

Causes for surface texture on processed sheet metal parts are microtexture of the material itself, resulting glints, shadows as well as imperfection introduced by the forming process (e.g., deep drawing) [3]. Surfaces with poor 3D features are sparsely textured, since there is minor curvature and thus few shadows. Also, sheet metal processing can be handled well and few imperfections are introduced. Glints are mostly dependend on camera and projector orientation, however spurious features are taken into account to an extent by using more than one camera for 3D measurement (see [3]).

### 3.2. 2D Keypoints and Matching

Due to the sparseness of texture information, it is reasonable to treat any occurrence of texture as a keypoint. A keypoint is defined in its corresponding 2D image coordinate system $\left(u_{c},v_{c}\right)$ and designated by the symbol $\left(u_{i,c}^{K},v_{i,c}^{K}\right)$ in the following (meaning a keypoint found in the *c*th camera of the *i*th measurement). Keypoint detection is comprised of texture binarisation with a threshold and computing centroids of the resulting connected components. Only connected components exceeding a minimum area are considered.

Because of the sparse texture properties, most feature descriptors fail to give distinct local characterisations. Block matching is thus carried out for regions of size $\left[\left(2u_{r}+1\right),\left(2v_{r}+1\right)\right]$ around keypoints and evaluated with the normalized cross correlation function. Parameters $u_{r},v_{r}>0$ are the pixel radius designating the size of correlation blocks. For two different measurements $i_{1}$ (*target*), $i_{2}$ (*source*) and corresponding cameras $c_{1},c_{2}$ it is given by

(7) $$\begin{array}{cc} f_{ncc}\left(u_{i_{1},c_{1}}^{K},v_{i_{1},c_{1}}^{K},u_{i_{2},c_{2}}^{K},v_{i_{2},c_{2}}^{K}\right) & =\\ & \frac{\sum_{t_{u}=-u_{r}}^{u_{r}}\sum_{t_{v}=-v_{r}}^{v_{r}}T_{i_{1},c_{1}}^{K}\left(t_{u},t_{v}\right)\;\cdot \;T_{i_{2},c_{2}}^{K}\left(t_{u},t_{v}\right)}{\sqrt{\left(\sum_{t_{u}=-u_{r}}^{u_{r}}\sum_{t_{v}=-v_{r}}^{v_{r}}{\left[T_{i_{1},c_{1}}^{K}\left(t_{u},t_{v}\right)\right]}^{2}\right)\;\cdot \;\left(\sum_{t_{u}=-u_{r}}^{u_{r}}\sum_{t_{v}=-v_{r}}^{v_{r}}{\left[T_{i_{2},c_{2}}^{K}\left(t_{u},t_{v}\right)\right]}^{2}\right)}} \end{array}$$

with the texture block surrounding a keypoint $\left(u_{i,c}^{K},v_{i,c}^{K}\right)$ defined by

(8) $$T_{i,c}^{K}\left(t_{u},t_{v}\right)=T_{i,c}\left(u_{i,c}^{K}+t_{u},v_{i,c}^{K}+t_{v}\right)$$

**Figure 6:**
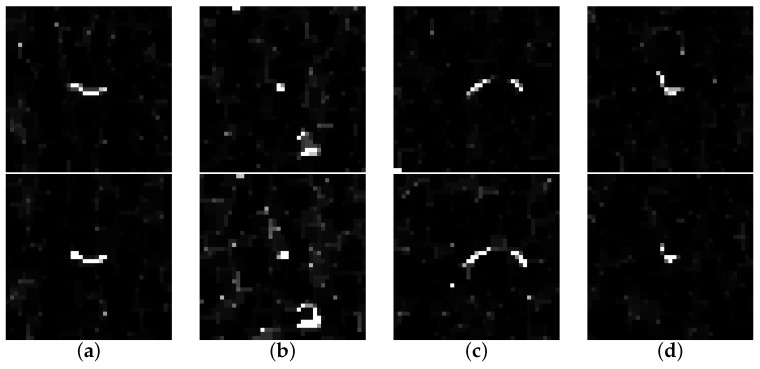
Example matched texture blocks (41 by 41 pixels) of measurement 2 (top) and 3 (bottom).

**Figure 7:**
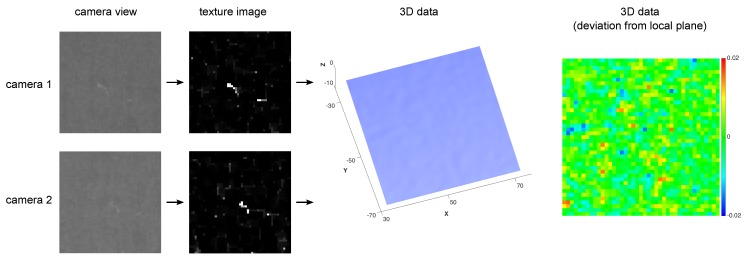
Segment around a keypoint used for blockmatching, given as camera view and texture image for the stereo pair, and corresponding 3D data. For better visibility, deviation of 3D data from a locally fitted plane is given color-coded (scale: +/- 20 µm).

The cross correlation is known to be robust against image noise; its normalisation gives further independence from local illumination variations [33,34]. The block size parameters $u_{r},v_{r}$ are chosen empirically by considering several properties. First, the distribution of areas of connected components is observed in the binarised texture used for keypoint detection. The pixel area occuring most frequently indicates the minimum block size necessary. Then, some blocks are auto-correlated within its corresponding image, to see if the correlation function in Equation (7) gives more than one maximum. Block size is then increased accordingly, in order to give a more unique result for block correlation. Several examples of sucessfully matched texture blocks of size $u_{r}=v_{r}=20$ pixels are given in Figure 6. Another example of a texture block during the various processing stages of the algorithm is given in Figure 7; in contrast to the texture, the 3D data does not yield any significant information.

Correlation is computed pairwise between the target and source measurements ($i_{1}\neq i_{2}$). By repeating on all possible pairs of cameras $c_{1},c_{2}$, a more robust matching of view-angle dependent surface features can be obtained. So for the assumed case of two measurements and a two-camera setup, this results in a total of four possible camera pairs $\left(c_{1},c_{2}\right)=\left\{\left(1,1\right),\left(1,2\right),\left(2,1\right),\left(2,2\right)\right\}$, for which block matching of all possible keypoint-combinations is computed. Only best matches are kept, which are indicated by a maximum of the correlation function $f_{ncc}$. Key points are also matched to be unique, *i.e*., each key point from a target texture is uniquely matched to a key point from source texture. If a key point from source texture is used multiple times, only the match with best correlation will be kept. The resulting total number of matched unique keypoints *κ* is usually a few hundreds.

### 3.3. Estimation of 3D Parameters

The result of the previous step is a set of 2D keypoints $K_{i1}=\left\{\;{\left(u_{i1,c}^{K},v_{i1,c}^{K}\right)}_{k}|k=1...\kappa \;\right\}$ given in measurement $i_{1}$, and a set of matching keypoints $K_{i2}=\left\{\;{\left(u_{i2,c}^{K},v_{i2,c}^{K}\right)}_{k}|k=1...\kappa \;\right\}$ in measurement $i_{2}$. As a prerequisite for 3D parameter estimation, the corresponding 3D keypoints have to be found.

Solving for the inverse camera calibration $f_{c}^{-1}\left(u_{i,c},v_{i,c}\right)$ is an underdetermined problem with one degree of freedom. In geometrical terms, this solution can be interpreted as the projection ray of the corresponding 2D image point. By intersecting the ray with the surface point cloud $P_{i}$, the matched 2D keypoint sets $K_{i1}$ and $K_{i2}$ are projected into the respective measurements’ 3D sensor coordinate system. A number of standard methods (line clipping algorithms) in computer graphics are available for this purpose [35]. This results in two sets of element-wise matching 3D keypoints $Q_{i_{1}}=\left\{\;q_{i_{1},k}\in R^{3}|k=1...\kappa \;\right\}$ and $Q_{i_{2}}=\left\{\;q_{i_{2},k}\in R^{3}|k=1...\kappa \;\right\}$ given in the sensor coordinate system of measurement $i_{1}$ and $i_{2}$, respectively.

The goal of the 3D parameter estimation is to find rotation matrices $R_{i_{1}},R_{i_{2}}$ and translation vectors $t_{i_{1}},t_{i_{2}}$ for $Q_{i_{1}}$ and $Q_{i_{2}}$ into a common world coordinate system, where the mean squared error (MSE) of the euclidean distance between matched point pairs is minimal. By arbitrarily chosing the world coordinate system (X,Y,Z) to be equal to the sensor coordinate system of one measurement $i_{1}$, we can describe the problem as the minimisation

(9) $$E\left(R_{i_{2}},t_{i_{2}}\right)=\sum_{k=1}^{\kappa }{\text{err}}_{i_{1},i_{2},k}^{2}\left(R_{i_{2}},t_{i_{2}}\right)\to \min_{R_{i_{2}},t_{i_{2}}}$$

of the squared keypoint error

(10) $${\text{err}}_{i_{1},i_{2},k}^{2}\left(R_{i_{2}},t_{i_{2}}\right)={\left[q_{i_{1},k}-\left(R_{i_{2}}\;\cdot \;q_{i_{2},k}+t_{i_{2}}\right)\right]}^{2}$$

The rotation and translation parameters $R_{i2},t_{i2}$ thus result from least-squares minimiziation of the summed squared error (which is the distance of a target keypoint to their corresponding transformed source keypoint). However, the sparseness of texture properties leads to ambiguities and a majority of falsely matched keypoints. In our experiments, around 70% of the *κ* matched keypoints are blunders.

In order to achive a robust selection of correct matches, we adapt the Random Sample Consensus (RANSAC [26]) method to our registration problem. Its main premise is that only an unknown subset of the *κ* candidates (so called *inliers*) fit our model of rigid registration.

**Figure 8:**
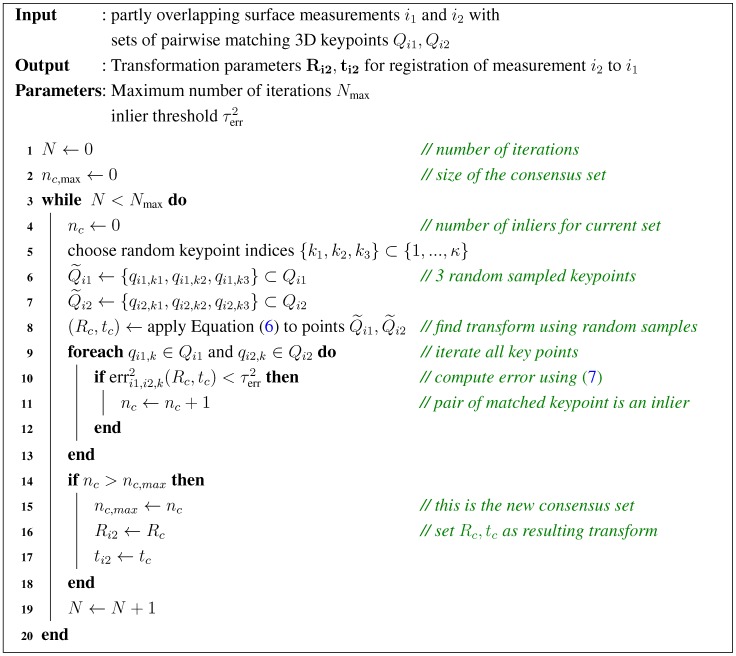
Pseudo-Code of the RANSAC algorithm.

If we consider the minimisation Equation (9), not all keypoint errors should be summed, but only those which fit the model. By defining an error threshold $\tau_{\text{err}}$, a pair of matched keypoints $\left(q_{i_{1},k},q_{i_{2},k}\right)$ is considered an *inlier* if the resulting squared error lies below the threshold $err_{i_{1},i_{2},k}^{2}<\tau_{\text{err}}^{2}$. Since the set of inliers is not known, we randomly select an inital set of three matched keypoints (the minimum number needed for computation) to find $R_{i_{2}}$ and $t_{i_{2}}$ from Equation (9). We then compute the error according to Equation (10) for all remaining matched keypoints, to find the size of the resulting inlier set. After repeating this random process a sufficient number of times, the largest set (consensus set) determines the correct transformation parameters. A pseudocode description of the algorithm is given in Figure 8.

## 4. Experimental Results and Discussion

The algorithm was tested with 5 partially overlapping measurement patches of a car door panel made of anodized aluminum, before painting and assembly. The resulting global registration of all 5 patches is shown in Figure 1, where each of the measurements is indicated by a coloured frame. Measurement data was acquired with a sensor consisting of two gray scale cameras and a DLP projector using a two-stage graycode and phase shift pattern. The cameras were arranged at a base distance of 540 mm and equipped with a f= 50 mm lens. This leads to a measurement area of approximately 300 mm by 400 mm with the depth resolution given as Δz=10 µm on a regular 1 mm by 1 mm *x*-*y* grid. Details of the camera calibration can be found in Table 1 and in the Appendix.

The most critical algorithm parameter is the block size for texture matching, which depends on texture properties and image scale. Large values are needed in order to minimize ambiguities, but heavily increase computation time. Auto-correlation can be used for determining the uniqueness of texture on an image with given keypoints. For the given measurement, a radius of $u_{r}=v_{r}=20$ pixels has been chosen, leading to texture blocks of 41 by 41 pixels (see Figure 6).

**Figure 9:**
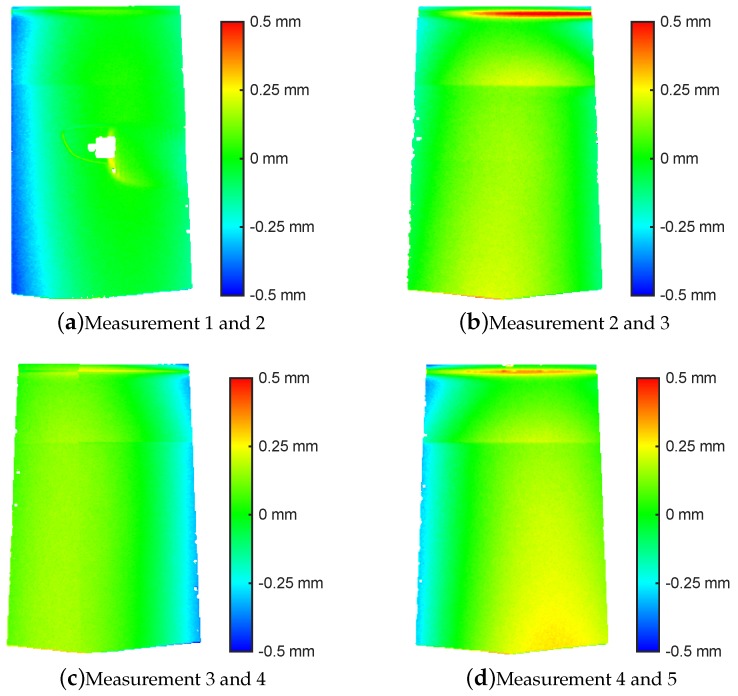
Point-to-plane distance (colour-coded) on overlap region of registered point clouds. Measurements 1, 2 and 3 correspond to the 3D data shown in Figure 1.

For applications in surface inspection, local surface quality is of importance, meaning that no local raggedness or imperfection of surface shape should be introduced by the registration algorithm. Thus a valid criterion for evaluation of the results is the pointwise euclidean distance on the overlap region. This is depicted as error maps in Figure 9, and summarized as minimum, maximum and mean squared error (MSE) for all registered pairs in Table 2.

As can be seen in the error maps (Figure 9), remaining errors lie within ±100 µm for most of the surface area, increasing along 3D-features like ridges as well as towards the margin areas. Maximum errors along the surface stay well below 1 mm. The low error along the majority of the measurement area is an encouraging result, especially considering that registration is based on selected keypoints rather than area optimisation.

If we compare all registered pairs (Table 2), it is obvious that most matched point pairs were found for measurement 1 and 2. Both measurements contain parts of very distinct 3D features (door handle cup), which is also reflected in the resulting 2D image features. The other patches contain few or ambiguous 3D features (e.g., design ridge), and thus rely completely on texture features. Typically, around 20 distinct texture areas are enough to get fairly good registration results. Further consideration of 3D shape may lead to better results. However typical 3D-based registration methods would fail in most cases without approximative registration parameters as given by our method.

An additional cause for increased margin errors are perspective distortions resulting from the imaging process. Despite the consideration in camera model and calibration, a systematic distortion increasing towards the outer areas of the measurement volume is a common effect in photogrammetric measurements. A possible solution would be weighting the two point clouds, thereby prefering 3D points which lie in the middle of the measurement area.

## 5. Conclusions

We have presented a method for registration of feature-poor 3D measurements from fringe projection systems based on matching of 2D texture features and a robust 3D-based model adjustment. Texture is extracted robust to uneven lighting and specular reflection caused by the projector light source. Image blocks are then correlated, which allows matching of delicate and sparse texture features that are typical to surfaces considered in inspection tasks. Prior knowledge of measurement position is not needed, allowing the automatic registration in semi-automatic and manual inspection applications.

The results are encouraging and yield information about sensor position and overlap areas of the measurement data. This is necessary for further local point-cloud based optimisation, e.g., with the Iterative Closest Point algorithm, which would fail on feature-poor surfaces without prior information.

## Figures and Tables

**Table 1:** Camera parameters of the calibrated sensor

Parameter		Camera A				Camera B		
t	[mm]	(266.4,	56.7,	829.2)		(−271.3,	57.0,	832.6)
$\left(\theta_{x},\theta_{y},\theta_{z}\right)$	[rad]	(−0.079,	0.311,	0.054)		(−0.077,	−0.320,	−0.053)
image size	[pixel]	1388 × 1038				1388 × 1038		
$\left(h_{u},h_{v}\right)$	[pixel]	(695.7,		494.1)		(678.6,		503.7)
*c*	[pixel]	2725.8				2721.0		
$s_{y}$		1.000				1.000		
$a_{1}$	[mm${}^{-2}$]	$-3.0\times 10^{-8}$				$-3.2\times 10^{-8}$		
$a_{2}$	[mm${}^{-4}$]	$8.4\times 10^{-15}$				$-8.2\times 10^{-15}$		

**Table 2:** Registration results for the overlapping regions of the considered 9 measurements: Number of total point pairs resulting from block matching, remaining consesus point pairs, and point-to-plane error within the overlapping area.

	# Point Pairs		Overlap Error		
Meas.	Matches	Consensus	Min [mm]	Max [mm]	MSE [mm]
1, 2	344	82	−0.457	0.333	0.105
2, 3	194	23	−0.549	0.701	0.096
3, 4	152	26	−0.599	0.417	0.139
4, 5	234	25	−0.451	0.457	0.142

## Appendix

### Camera Calibration Model

In the following section, we elaborate on the camera model for calibration of the sensor used in our experiments. Detailed characteristics of our calibrated camera system are given in Table 1.

- *External Calibration:*
  (11) $$\left(\begin{array}{c} x_{c}\\ y_{c}\\ z_{c} \end{array}\right)=R\;\cdot \;\left(\begin{array}{c} x\\ y\\ z \end{array}\right)-t$$
  $$\begin{array}{cc} \left(x,y,z\right) & 3\text{D world coordinates}\;(mm)\\ \left(x_{c},y_{c},z_{c}\right) & 3\text{D local sensor coordinates}\;(mm)\\ R & 3\times 3\;\text{rotation matrix},\text{characterised by rotation angles}\;(\theta_{x},\theta_{y},\theta_{z})\\ t & 3\text{D translation vector}\;(mm) \end{array}$$
- *2D Projection:*
  (12) $$\left(\begin{array}{c} u\\ v \end{array}\right)=\frac{c\;\cdot \;d_{r}}{z_{c}}\left[\begin{array}{cc} 1 & 0\\ 0 & s_{y} \end{array}\right]\left(\begin{array}{c} x_{c}\\ y_{c} \end{array}\right)+\left(\begin{array}{c} h_{u}\\ h_{v} \end{array}\right)$$
  $$\begin{array}{cc} \left(u,v\right) & 2\text{D image coordinates}\;(pixels)\\ c & \text{focal length}\;(pixels)\\ d_{r} & \text{radial distortion}\;(\text{see below})\\ s_{y} & \text{pixel}\;x/y\;\text{scale}\\ \left(h_{u},h_{v}\right) & \text{principal point}\;(pixels) \end{array}$$
- *Radial Distortion:*
  (13) $$d_{r}=1+a_{1}\left(r^{2}-r_{0}^{2}\right)+a_{2}\left(r^{4}-r_{0}^{4}\right)$$
  (14) $$\text{with}\;r^{2}=x_{c}^{2}+y_{c}^{2}$$
  $$\begin{array}{cc} a_{1},a_{2} & \text{first},\text{second order radial distortion coefficients}\\ r & \text{radial coordinate component}\\ r_{0} & \text{radial coordinate zero-crossing} \end{array}$$
